# Resistance training combined with blood flow restriction in cirrhosis: study protocol for a randomized controlled trial

**DOI:** 10.1186/s13063-020-04410-2

**Published:** 2020-05-29

**Authors:** Sanmy Rocha Nóbrega, Silvana Gama Florencio Chachá, Cleiton Augusto Libardi

**Affiliations:** 1 MUSCULAB - Laboratory of Neuromuscular Adaptations to Resistance Training, Federal University of São Carlos – UFSCar, Rod. Washington Luiz, km 235 – SP 310, São Carlos, SP CEP 13565-905 Brazil; 2 Department of Medicine, Federal University of São Carlos – UFSCar, Rod. Washington Luiz, km 235 – SP 310, São Carlos, SP CEP 13565-905 Brazil

**Keywords:** End stage liver disease, Sarcopenia, Physical activity, Muscle strength, Quality of life, Hypertrophy

## Abstract

**Background:**

Patients affected by hepatic cirrhosis show reductions in muscle mass and function, with poor quality of life and functional performance. As such, resistance training with blood flow restriction (BFR-RT) could be a useful therapeutic tool for health promotion. Thus, we aim to verify the effects of this intervention on muscle strength, muscle mass, fiber Pennation angle, fascicle length, functional performance, quality of life, and fall risk scores in this population.

**Methods:**

Thirty participants will be randomly distributed between 1) BFR-RT and 2) control (CTRL). Assessments will occur at three time points: before the training intervention (0 W), after 12 weeks (12 W), and at follow-up (24 W). The following variables will be assessed: Child-Pugh classification; MELD score; SF-36 questionnaire; fatigue severity index; 6-min walk test; timed-up and go; 30-s sitting and rising test; dietary record; one-repetition maximum (1-RM) strength test (knee extension exercise); and vastus lateralis’ cross-sectional area, Pennation angle, and fascicle length. The BFR-RT group will undergo 12 weeks of knee extension exercise (1 × 30 repetitions and 3 × 15 repetitions at 20% 1-RM and 50% of total blood flow occlusion pressure), with two sessions per week. Data normality will be assessed using the Shapiro-Wilk test. In case of normal distribution, a one-way repeated measures analysis of variance will be implemented to test for differences in baseline values. A mixed model then will be applied for each dependent variable. In case of non-normal data distribution, a Kruskal–Wallis test will be implemented to test for differences in baseline values. Next, the Friedman test will be used to analyze repeated measures. Within- and between-group effect sizes will be calculated using Cohen’s *d* for each outcome. Finally, the minimal clinically important difference will be analyzed with distribution-based methods.

**Discussion:**

To our knowledge, this will be the first trial to investigate BFR-RT in patients with cirrhosis and evaluate the effects on neuromuscular parameters, functional performance, disease severity, and quality of life outcomes.

**Trial registration:**

Brazilian Clinical Trials Registry (ReBec): RBR-395mfw. Registered on 25 August 2018.

## Background

Liver cirrhosis (LC) is considered the final outcome of chronic liver injury and is characterized by the development of diffuse regenerative nodules enveloped in fibrous tissue and progressive hepatic dysfunction [1, 2]. Many different complications can be associated with LC. One important implication for the cirrhotic patient is sarcopenia [3], a syndrome characterized by muscle wasting and loss of muscle strength [4]. Different factors have been associated with sarcopenia onset in LC; one is the reduced physical activity level commonly observed in this population [5, 6]. As a result, sarcopenia causes even further reductions in physical activity levels, as well as reduced quality of life, decline in functional performance, increase in fall risk, development of other complications of LC (e.g., sepsis-related death, uncontrolled ascites, hepatic encephalopathy), poor outcomes after liver transplantation, and reduction in survival rate [7–9]. Thus, a physical activity program has been proposed to help attenuate sarcopenia impact in LC, ultimately improving quality of life and survival [9].

In this sense, for the general population, resistance training (RT), either at high-load (~ 80% one-repetition maximum (1-RM)) or at low-load (~ 20–30% 1-RM) to muscle failure, is well known to result in marked increases in muscle strength and mass [10], which can be accompanied by modifications in muscle architecture (e.g., fiber Pennation angle and fascicle length [11]). Additionally, improvements in functional performance [12] and quality of life [13] also have been reported. Considering sarcopenia occurrence in LC and the effects of RT on muscle strength and mass, RT could be an effective therapeutic tool in LC patients for both prevention and attenuation of sarcopenia. However, high-load RT and low-load RT to muscle failure may not be safe for the individual with LC. Even moderate exercise can increase hepatic venous pressure gradient of cirrhotic patients [14] and is associated with greater risk of variceal bleeding, impairing exercise practice.

In this sense, evidence shows that low-load RT combined with partial blood flow restriction (BFR-RT) results in significant muscle mass gains, comparable to high-load RT [15, 16] or low-load RT to muscle failure [17]. Its applicability in frail populations (e.g., elderly and cardiac patients) also has been verified, with positive outcomes after the intervention period [15, 18, 19], including functional performance [20]. Additionally, BFR-RT has been found to result in significantly smaller increases in systolic blood pressure, diastolic blood pressure and heart rate than both low-load RT and high-load RT performed to failure [21]. Thus, BFR-RT could prove to be a safe RT method in LC patients, improving muscle strength and mass while avoiding marked increases in blood pressure and, possibly, in portal pressure and variceal bleeding.

Thus, this study aims to investigate the effects of BFR-RT on muscle strength, muscle mass, fiber Pennation angle, fascicle length, functional performance, and risk of falls in LC patients. Additionally, we aim to verify whether BFR-RT is a safe method for this population. Our hypothesis is that BFR-RT will promote increases in muscle strength, muscle mass, and functional performance, while reducing the risk of falls, with no aggravation in LC condition.

## Methods/Design

This study is registered with Clinical Trials Brazil (http://www.ensaiosclinicos.gov.br), registration number RBR-395mfw. All study procedures were approved by the institution’s Ethics Committee.

### Aims

The aims of this study are as follows:

- Verify the effects of BFR-RT on the knee extensor strength of LC patients
- Verify the effects of BFR-RT on the vastus lateralis muscle cross-sectional area, fiber Pennation angle, and fascicle length
- Verify the effects of BFR-RT on the functional performance and risk of falls in LC patients
- Verify the retention of BFR-RT effects after 12 weeks of protocol cessation (24 weeks)
- Verify whether BFR-RT results in adverse events in LC patients

### Design overview

**Fig. 1:**
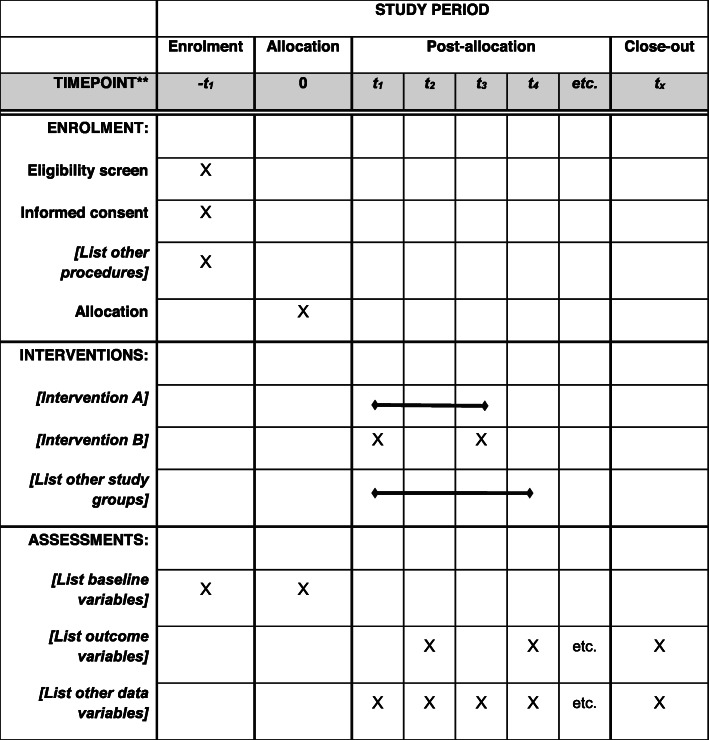
Template of recommended content for the schedule of enrollment, interventions, and assessments

**Fig. 2:**
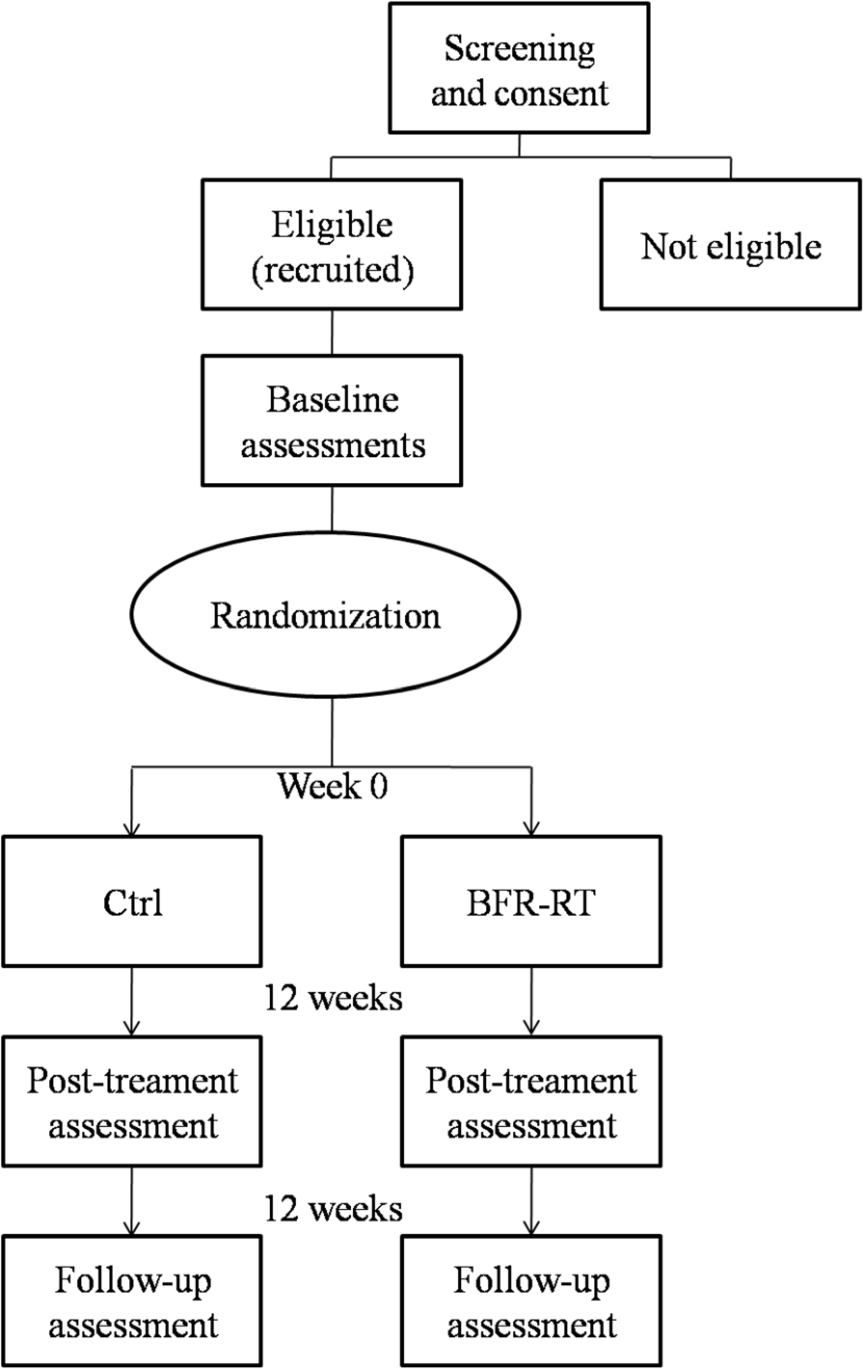
Design overview. Before the training period (0 W), all participants will be screened for Child-Pugh class and MELD score, as well as for quality of life, functional capacity, and risk of falls Participants will also be requested to provide a dietary record for caloric intake calculation. At least 48 h later, participants will perform a 1-RM test. Then 72 h later, vastus lateralis muscle CSA, PA, and FL will be assessed. Participants will then be randomly allocated between a training (resistance training with blood-flow restriction - BFR-RT) and control (CTRL) group. After 12 weeks of intervention (12 W), all of the previous parameters will be reassessed. A no-intervention period of 12 weeks will be carried out and, at follow-up (24 W), participants will be reassessed in order to verify the interventions carryover effects

This study is an experimental approach with repeated measures for data collection to investigate the efficacy and safety of a BFR-RT protocol in LC patients (Additional file 1 - SPIRIT 2013 Checklist) (Fig. 1). All participants (target *n* = 30) will be evaluated at three time points: before the training period (0 W), after 12 weeks (12 W), and at follow-up (24 W). Participants will be randomly allocated between a training (BFR-RT) and control (CTRL) group. Both groups will undergo their respective interventions two times per week for 12 weeks, followed by a no-intervention period of 12 weeks to verify the interventions carryover effects (Fig. 2).

### Setting

**Fig. 3:**
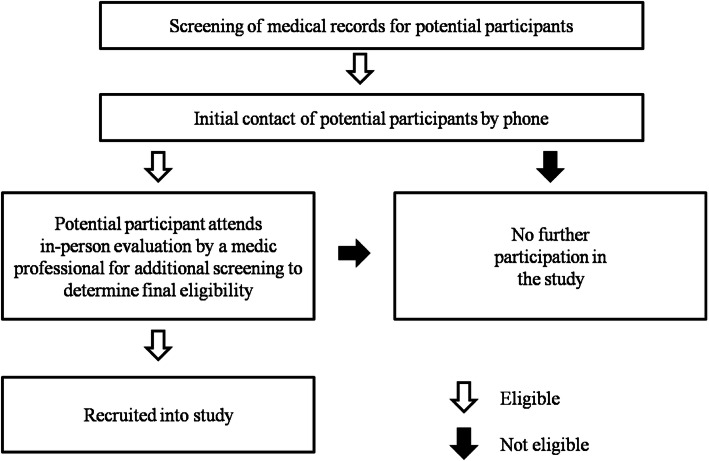
Participant recruitment steps

Recruitment will be carried out at the University Hospital and at *CAIC* (Chronic Infection Care Center). Medical records will be screened for preliminary eligibility. Participants who meet initial eligibility criteria will undergo in-person evaluation by a medic professional specialized in liver disease and will be requested to provide an informed consent before study initiation (Fig. 3). All experimental procedures will be carried out at the University Hospital.

### Eligibility criteria

Eligibility criteria are 1) liver cirrhosis diagnosed by histopathological evaluation, hepatic elastography, or by clinic, radiologic or endoscopic criteria and 2) age ≥ 18 and ≤ 70 years. Exclusion criteria are 1) less than 6 months of alcohol abstinence, 2) MELD score > 15, 3) Child-Pugh class C, 4) decompensated chronic diseases, 5) diabetes mellitus, 6) hepatocellular carcinoma or others neoplasia, 7) previous liver transplant, 8) severe ascites, 9) persistent hepatic encephalopathy, 10) coagulation disorders characterized as INR ≥ 1.5 or platelets ≤ 75.000, or 11) medical contraindications for physical exercise practice.

### Randomization

Initially, all participants will be pooled into a single group. This group then will be organized into quartiles according to participants’ initial 1-RM and vastus lateralis CSA. Next, participants in each quartile will be allocated to either the training (BFR-RT) or control group (CTRL) using a maximally tolerated imbalance model [22]. An unpaired T test will be applied to ensure no baseline difference between groups. If significant between-groups differences are detected, the randomization procedure will be repeated until a random distribution with no significant difference is achieved. The Berger-Exner test will be performed during data analysis to verify for selection bias [23].

## Study interventions

### Resistance training with blood-flow restriction (BFR-RT)

Blood-flow restriction pressure will be determined before initiation of the training protocol. Participants will be asked to rest comfortably in supine position for approximately 10 min. A vascular Doppler probe (DV-600; Marted, Ribeirão Preto, São Paulo, Brazil) then will be placed over the tibial artery to capture its auscultatory pulse. A standard blood pressure cuff (175 × 94 mm, JPJ Industria Comercio Assistencia Técnica Mat Hospitalar Ltda Me, Sao Paulo, Brazil) will be attached to the participant’s quadriceps (near the inguinal fold region) and inflated to achieve auscultatory pulse interruption [24]. The restriction pressure used in the BFR-RT group will be determined as 50% of the pressure necessary for complete auscultatory pulse interruption in the resting condition [15].

The training protocol will consist of bilateral 1 × 30 repetitions and 3 × 15 repetitions at 20% 1-RM associated with blood flow restriction [15] in a knee extension machine (FISIOMAQ, Paraná, Brazil). The training session will start with a general warm-up on a cycloergometer (Ergo-Fit®, Pirmasens, Rheinland-Pfalz, Germany) at 20 km·h^− 1^ for 5 min. Next, the cuff (175 × 94 mm) will be placed proximally on the thigh (inguinal fold region) and inflated to 50% of complete blood flow restriction. The cuff will remain inflated throughout the exercise but will be released in the rest intervals. After the 6th training week, intensity will be increased to 30% 1-RM. A 2-min rest will be allowed between sets.

### Control group (CTRL)

The relaxation protocol will serve as a sham intervention in order to avoid possible effects from attention and contact time with the researchers [25]. The protocol will consist of 30-min sessions, including cephalocaudal muscle relaxation and breathing exercises.

## Assessments

### Primary outcomes

#### Maximal dynamic strength test (1-RM)

Maximal dynamic strength will be tested using one-repetition maximum tests (1-RM). Bilateral 1-RM tests will be performed on a knee extension machine, according to the protocol of Brown and Weir [26]. Initially, a warm-up of 5 min will be performed on a cycloergometer at 20 km·h^− 1^. Then, a specific warm-up for the assessed muscles will be performed using eight repetitions, followed by three repetitions at 50% and 70% of an estimated 1-RM, respectively. A rest interval of 2 min will be allowed between sets. Following warm-up, the 1-RM test will be initiated. Participants will perform both concentric and eccentric phases of the movement. An attempt will be considered successful if complete knee extension (~ 170°) is achieved. Up to five attempts will be allowed, with a rest interval of 3 min between attempts.

#### Muscle cross-sectional area (CSA)

Vastus lateralis (VL) muscle cross-sectional area (CSA) will be assessed using an ultrasound (US) machine. Procedures similar to Lixandrão et al. [27] will be adopted. Participants will be instructed to abstain from vigorous physical activities for at least 72 h. Following their arrival, participants will lay in a supine position for 15 min to allow fluid shifts to occur. Images will be collected using the US B-mode with a 7.5 MHz probe (Samsung, MySono U6, industria e comércio Ltda. São Paulo, Brazil). Surface gel will be applied to promote acoustic coupling while avoiding dermal deforming. The distance between the greater trochanter and the lateral epicondyle will be manually calculated, and the CSA will be obtained at the 50% point. Sequential markings will be made on participants’ skin to guide probe movement, and images will be acquired every 2 cm. Following data acquisition, VL CSA will be reconstructed according to Reeves et al. [28], whereby images will be sequentially opened and rotated using PowerPoint version 2007 (Microsoft, Redmond, WA, USA), until the full muscle area is visible. The CSA value will be assessed using the ImageJ polygonal tool. Each muscle area will be reconstructed two times, and the mean will be considered the true CSA value.

### Secondary outcomes

#### Child-Pugh class and MELD score

Participant blood samples will be collected and analyzed in a professional clinical laboratory. To classify these samples according to Child-Pugh class and Meld score at 0 W, 12 W and 24 W, they will be screened for aspartate aminotransferase, alanine aminotransferase, creatinine, albumin, and bilirubin. Additionally, clinical screenings will be carried out by a medical professional at the same time points for physical assessment of the participants.

### Quality of life assessment

Participant quality of life will be assessed using both SF-36 questionnaire and fatigue severity scale. A researcher properly familiarized with both tools will perform these assessments.

### Dietary record

Each participant’s food intake in the previous 24 h will be investigated and recorded for later analysis. A nutritionist familiarized with the procedure will perform assessments at all time points. Dietary record data will be analyzed using DietWin 2012 (DietWin Software de Nutrição, Porto Alegre, Rio Grande do Sul, Brazil) professional software.

### Timed up and go test (TUG)

Initially, participants will be positioned sitting on a chair. Then, they will be requested to stand up, walk 3 m, come back, and sit down with no exterior help [29]. Each participant will be timed throughout the trial. These trials will be repeated two times, and the average between trials will be considered for analysis.

### Six-minute walk test (6MWT)

The test will be performed following the American Thoracic Society guideline [30]. In short, participants will be asked to walk back and forth for 6 min along a 30-m corridor marked every 3 m. After 6 min, the total walk distance will be calculated.

### Sitting-rising test (SRT)

The test will be initiated with participants sitting in a chair. Participants will be requested to sit in an upright position with both feet firmly touching the ground and arms crossing their chest. At the researcher signal, the participant will get up and sit down, returning to the initial position. Participants will be encouraged to get up and sit down the maximum number of times in a 30-s period. Only repetitions properly executed will be counted [31].

### Pennation angle (PA)

VL PA will be assessed using B-mode ultrasound at the point corresponding to 50% of the femur’s length. The transducer will be placed longitudinally to muscle tissue and, when necessary, will be laterally tilted to better allow fascicle visualization [32]. Muscle fiber PA will be determined as the intersection of the fascicles with the deep aponeurosis, as assessed with ImageJ angular tool. Each image will be analyzed twice, and the mean value between both analyses will be considered as PA.

### Fascicle length (FL)

B-mode ultrasound will be used for FL assessment at the same site used for PA, with the same probe placement. Linear extrapolation will be used to calculate FL whenever a fascicle extends itself beyond the ultrasound field of view [32]. FL will be calculated two times per image. The mean value will be used as true FL.

## Data analysis

Following visual inspection, data normality will be assessed using the Shapiro-Wilk test. In case of normal distribution, a one-way repeated measures analysis of variance (ANOVA) will be implemented to test for differences in baseline values. Then, a mixed model will be applied for each dependent variable (Child-Pugh class, MELD score, SF-36 and FSS scores, dietary records, TUG, 6MWT, SRT, 1-RM, CSA, PA and FL), having group (BFR-RT and CTRL) and time (0 W, 12 W and 24 W) as fixed factors, and participants as random factors. In case of non-normal data distribution, a Kruskal–Wallis test will be implemented to test for differences in baseline values. Then, the Friedman test will be used to analyze repeated measures. To avoid multiplicity interference on data analysis, adjusted *p* values will be calculated for all variables. Adjusted *p* values will be calculated using Hommel’s procedure, and both adjusted and non-adjusted values will be reported. Additionally, within-group (0-12 W and 0-24 W changes) and between-group effect sizes will be calculated using Cohen’s *d* [33] for each outcome. Finally, the minimal clinically important difference will be analyzed with distribution-based methods [34]. Statistical analysis will be carried out using SAS 9.3 software (SAS institute Inc., Cary, NC).

## Discussion

LC was reported as the 14th most common cause of death worldwide in 2012, resulting in 1.03 million deaths per year [35], with sarcopenia affecting up to 70% of the patients afflicted by LC [3]. If proven efficient in countering sarcopenia onset or reducing sarcopenia progression, new therapeutic options (e.g., BFR-RT) could help reduce the treatment costs of patients with LC.

As such, the present study aims to verify whether BFR-RT will result in important improvements in muscle strength and mass in patients with liver cirrhosis, as well as verifying any significant differences in functional performance and risk of falls. In addition, the LC Child-Pugh class and MELD score will be evaluated in order to determine BFR-RT safety and effects on the disease’s symptoms. To our knowledge, this will be the first intervention study to investigate BFR-RT in this population. Considering that current evidence shows BFR-RT is a safe method, even for frail populations [15, 17, 18], we believe BFR-RT will result in important increases in muscle mass and strength, accompanied by increases in functionality, with no negative outcomes on cirrhosis condition. There are some limitations to this study. The limited follow-up period prevents us from drawing conclusions about the long-term effects of the proposed intervention. Second, we opted to limit our sample to patients without severe LC complications (i.e., Child-Pugh class B and C and MELD > 15), limiting our ability to generalize our results to patients with decompensated LC.

## Trial status

This is version 1 of this protocol, written on July 13, 2018. Participant recruitment began in September 2018. Recruitment was expected to be completed on October 2018; however, an insufficient number of participants were recruited. Thus, recruitment is expected to be completed in July 2020. Recruitment was ongoing at the time of submission.

## Supplementary information

**Additional file 1.** SPIRIT 2013 Checklist.

## Data Availability

Data sharing is not applicable to this article as no datasets were generated or analyzed during the current study.

## Abbreviations

1-RM: One-repetition maximum
6MWT: Six-minute walk test
ANOVA: Analysis of variance
BFR-RT: Blood-flow restriction resistance training
CAIC: Chronic infection care center
CTRL: Control group
CSA: Cross-sectional area
FL: Fascicle length
FSS: Fatigue severity scale
INR: International normalized ratio
LC: Liver cirrhosis
PA: Pennation angle
RT: Resistance training
SRT: Sitting-rising test
TUG: Timed up and go
US: Ultrasound
VL: vastus lateralis
